# Immunohistochemical characteristics of local sites that trigger atrial arrhythmias in response to high-frequency stimulation

**DOI:** 10.1093/europace/euac176

**Published:** 2022-10-19

**Authors:** Min-young Kim, James Nesbitt, Simos Koutsoftidis, Joseph Brook, David S Pitcher, Chris D Cantwell, Balvinder Handa, Catherine Jenkins, Charles Houston, Stephen Rothery, Anand Jothidasan, Justin Perkins, Poppy Bristow, Nick W F Linton, Emm Drakakis, Nicholas S Peters, Rasheda A Chowdhury, Prapa Kanagaratnam, Fu Siong Ng

**Affiliations:** Myocardial Function Section, National Heart and Lung Institute, Imperial College London, Du Cane Road, London W12 0NN, UK; Department of Cardiology, Hammersmith Hospital, 72 Du Cane Rd, London, W12 0HS, UK; Imperial Centre for Cardiac Engineering, Imperial College London, Level 2, Faculty Building, South Kensington Campus, London SW7 2AZ, UK; Myocardial Function Section, National Heart and Lung Institute, Imperial College London, Du Cane Road, London W12 0NN, UK; Department of Cardiology, Hammersmith Hospital, 72 Du Cane Rd, London, W12 0HS, UK; Imperial Centre for Cardiac Engineering, Imperial College London, Level 2, Faculty Building, South Kensington Campus, London SW7 2AZ, UK; Myocardial Function Section, National Heart and Lung Institute, Imperial College London, Du Cane Road, London W12 0NN, UK; Imperial Centre for Cardiac Engineering, Imperial College London, Level 2, Faculty Building, South Kensington Campus, London SW7 2AZ, UK; Myocardial Function Section, National Heart and Lung Institute, Imperial College London, Du Cane Road, London W12 0NN, UK; Imperial Centre for Cardiac Engineering, Imperial College London, Level 2, Faculty Building, South Kensington Campus, London SW7 2AZ, UK; Myocardial Function Section, National Heart and Lung Institute, Imperial College London, Du Cane Road, London W12 0NN, UK; Imperial Centre for Cardiac Engineering, Imperial College London, Level 2, Faculty Building, South Kensington Campus, London SW7 2AZ, UK; Myocardial Function Section, National Heart and Lung Institute, Imperial College London, Du Cane Road, London W12 0NN, UK; Department of Cardiology, Hammersmith Hospital, 72 Du Cane Rd, London, W12 0HS, UK; Imperial Centre for Cardiac Engineering, Imperial College London, Level 2, Faculty Building, South Kensington Campus, London SW7 2AZ, UK; Myocardial Function Section, National Heart and Lung Institute, Imperial College London, Du Cane Road, London W12 0NN, UK; Imperial Centre for Cardiac Engineering, Imperial College London, Level 2, Faculty Building, South Kensington Campus, London SW7 2AZ, UK; Myocardial Function Section, National Heart and Lung Institute, Imperial College London, Du Cane Road, London W12 0NN, UK; Imperial Centre for Cardiac Engineering, Imperial College London, Level 2, Faculty Building, South Kensington Campus, London SW7 2AZ, UK; Myocardial Function Section, National Heart and Lung Institute, Imperial College London, Du Cane Road, London W12 0NN, UK; Imperial Centre for Cardiac Engineering, Imperial College London, Level 2, Faculty Building, South Kensington Campus, London SW7 2AZ, UK; The Facility for Imaging by Light Microscopy, Sir Alexander Fleming Building, South Kensington Campus, Imperial College London, Exhibition Road, London SW7 2AZ, UK; Department of Cardiothoracic Surgery, Royal Brompton and Harefield NHS Foundation Trust, 1 Manresa Rd, London SW3 6LR, UK; Royal Veterinary College, 4 Royal College St, London NW1 0TU, UK; Royal Veterinary College, 4 Royal College St, London NW1 0TU, UK; Myocardial Function Section, National Heart and Lung Institute, Imperial College London, Du Cane Road, London W12 0NN, UK; Department of Cardiology, Hammersmith Hospital, 72 Du Cane Rd, London, W12 0HS, UK; Imperial Centre for Cardiac Engineering, Imperial College London, Level 2, Faculty Building, South Kensington Campus, London SW7 2AZ, UK; Imperial Centre for Cardiac Engineering, Imperial College London, Level 2, Faculty Building, South Kensington Campus, London SW7 2AZ, UK; Myocardial Function Section, National Heart and Lung Institute, Imperial College London, Du Cane Road, London W12 0NN, UK; Department of Cardiology, Hammersmith Hospital, 72 Du Cane Rd, London, W12 0HS, UK; Imperial Centre for Cardiac Engineering, Imperial College London, Level 2, Faculty Building, South Kensington Campus, London SW7 2AZ, UK; Myocardial Function Section, National Heart and Lung Institute, Imperial College London, Du Cane Road, London W12 0NN, UK; Imperial Centre for Cardiac Engineering, Imperial College London, Level 2, Faculty Building, South Kensington Campus, London SW7 2AZ, UK; Myocardial Function Section, National Heart and Lung Institute, Imperial College London, Du Cane Road, London W12 0NN, UK; Department of Cardiology, Hammersmith Hospital, 72 Du Cane Rd, London, W12 0HS, UK; Imperial Centre for Cardiac Engineering, Imperial College London, Level 2, Faculty Building, South Kensington Campus, London SW7 2AZ, UK; Myocardial Function Section, National Heart and Lung Institute, Imperial College London, Du Cane Road, London W12 0NN, UK; Department of Cardiology, Hammersmith Hospital, 72 Du Cane Rd, London, W12 0HS, UK; Imperial Centre for Cardiac Engineering, Imperial College London, Level 2, Faculty Building, South Kensington Campus, London SW7 2AZ, UK

**Keywords:** Ganglionated plexus, Ganglionated plexi, Atrial fibrillation, Autonomic nervous system, Cardiac nervous system, Langendorff

## Abstract

**Aims:**

The response to high frequency stimulation (HFS) is used to locate putative sites of ganglionated plexuses (GPs), which are implicated in triggering atrial fibrillation (AF). To identify topological and immunohistochemical characteristics of presumed GP sites functionally identified by HFS.

**Methods and results:**

Sixty-three atrial sites were tested with HFS in four Langendorff-perfused porcine hearts. A 3.5 mm tip quadripolar ablation catheter was used to stimulate and deliver HFS to the left and right atrial epicardium, within the local atrial refractory period. Tissue samples from sites triggering atrial ectopy/AF (ET) sites and non-ET sites were stained with choline acetyltransferase (ChAT) and tyrosine hydroxylase (TH), for quantification of parasympathetic and sympathetic nerves, respectively. The average cross-sectional area (CSA) of nerves was also calculated. Histomorphometry of six ET sites (9.5%) identified by HFS evoking at least a single atrial ectopic was compared with non-ET sites. All ET sites contained ChAT-immunoreactive (ChAT-IR) and/or TH-immunoreactive nerves (TH-IR). Nerve density was greater in ET sites compared to non-ET sites (nerves/cm^2^: 162.3 ± 110.9 vs. 69.65 ± 72.48; *P* = 0.047). Overall, TH-IR nerves had a larger CSA than ChAT-IR nerves (µm^2^: 11 196 ± 35 141 vs. 2070 ± 5841; *P* < 0.0001), but in ET sites, TH-IR nerves were smaller than in non-ET sites (µm^2^: 6021 ± 14 586 vs. 25 254 ± 61 499; *P* < 0.001).

**Conclusions:**

ET sites identified by HFS contained a higher density of smaller nerves than non-ET sites. The majority of these nerves were within the atrial myocardium. This has important clinical implications for devising an effective therapeutic strategy for targeting autonomic triggers of AF.

**What’s new?**

- AF-triggering sites were identified by stimulating the intrinsic cardiac nerves in a decentralised porcine heart, evidence that the intrinsic cardiac autonomic nervous system can function independently of the extrinsic cardiac nervous system.
- Local atrial sites that triggered atrial arrhythmia with high-frequency stimulation contained a higher density of smaller nerves (both parasympathetic and sympathetic) than local sites that did not trigger atrial arrhythmias.
- The majority of the nerves in atrial arrhythmia triggering sites were in the myocardium. These findings have important clinical implications on devising an effective therapeutic strategy to map and target the autonomic triggers of AF.

## Introduction

The intrinsic cardiac autonomic nervous system (ANS) comprises a complex and interconnected network of ganglionated plexuses (GPs). Each ganglion contains densely packed neurones, and the GPs have the capability of independently functioning within their own local circuit, without the influence of the extrinsic cardiac ANS.^1^ GPs have been implicated in atrial arrhythmogenesis, with stimulation of the GPs leading to neurotransmitter release, such as acetylcholine and catecholamine, which can shorten the local action potential and lead to atrial arrhythmias.^2^

High-frequency stimulation (HFS) is a technique used to functionally identify GP sites by observing for specific arrhythmogenic responses following stimulation. Short bursts of HFS synchronized to the atrial stimulus and delivered within the local atrial refractory period to trigger atrial arrhythmias can identify the ectopy-triggering GP (ET-GP). ET-GPs occupy a discrete areas of the left atrium of the heart and have an important role in triggering atrial fibrillation (AF).^3^

In the clinical setting, the gold standard treatment for AF is wide antral circumferential ablation around all pulmonary veins (PV) to achieve complete electrical PV isolation (PVI).^4^ Recent large prospective randomized controlled trials have shown a success rate of 50–75% at 12 months for PVI.^5,6^ These results were comparable to the GANGLIA-AF (Ectopy-triggering ganglionated plexuses ablation to prevent atrial fibrillation) trial, where ET-GPs were mapped using HFS and ablated *without* PVI in patients with paroxysmal AF and showed 58% freedom from atrial arrhythmias at 12 months’ follow-up.^7^ However, it is important to note that HFS identifies *presumed* GP sites by utilizing their functional response to stimulation. There may be unique topological characteristics that are signatures for GP arrhythmogenic response to HFS that are yet to be established. Therefore, we sought to study the local tissue characteristics at sites that trigger atrial ectopy or arrhythmia with HFS, using an *ex vivo* porcine model, that closely represents the physiology and anatomy of a human heart.

## Methods

All animal studies were ethically reviewed and carried out in accordance with ethical standards (European Commission 2010). The protocol was approved by the Royal Veterinary College (RVC) Animal Welfare and Ethical Review Board. All animals were housed and transported under conditions specified in the UK’s Animal Welfare Act 2006 and The Welfare of Farm Animals (England) Regulations 2007. Animal care, investigations, and euthanasia were performed according to the Animals (Scientific Procedures) Act 1986, which is regulated by Schedule 1 of the Act. In all our anaesthetized animals, an overdose of anaesthetic agents was used while being unconscious to minimize distress.

### *In vivo* pig preparation for heart explantation

Four healthy white female pigs (weight 70–80 kg, age 4–5 months) were anaesthetized for the purpose of this study. Premedication included ketamine 20 mg/kg and midazolam 0.5 mg/kg injected intramuscularly (IM). Following premedication, a 22 gauge intravenous (IV) catheter was placed in the auricular vein and oxygen was supplied via a tight mask. Anaesthesia was induced with IV administration of propofol until intubation and the animals were connected to the anaesthetic machine (Carestastion 650, GE Healthcare, UK). Anaesthesia was maintained with sevoflurane (SevoFlo, Zoeitis, UK) vaporized in a mixture of oxygen and medical air. The end-tidal concentration of sevoflurane was maintained at 2.2–2.5%. This was monitored continuously and recorded every 5 min. The animals were positioned dorsally, and a catheter (Leadercath, Vygon, UK) was placed in the femoral artery for continuous invasive blood pressure monitoring. Analgesia was provided by a continuous infusion of fentanyl at a rate of 0.2 mcg/kg/min following an initial loading dose of 2 mcg/kg IV. All the animals were receiving fluid therapy of compound sodium lactate (CSL) solution at 5 mL/kg/hr IV, throughout the procedure. Monitoring was performed through a multiparameter monitor (Carestation 650, GE Healthcare, UK) and included anaesthetic gas analysis, capnography, electrocardiogram, oxygen saturation, temperature, and continuous invasive blood pressure measurement. At the end of the procedure, euthanasia was performed with an overdose of 0.7 mL/kg pentobarbital. Monitoring was continued until confirmation of her death.

### *Ex vivo* porcine whole heart preparation

Four whole hearts from the pigs were explanted and studied. All hearts had intact left atriums with one left and right PVs. The explanted porcine hearts from anaesthetized pigs were perfused with cardioplegia solution *in situ* then immediately submerged in a cardioplegia solution during transportation, up to a maximum of 90 min cold ischaemic time. Any non-cardiac tissue was carefully excised, leaving as much of the epicardial adipose tissue intact. The aorta was cut down to leave approximately 2 cm for cannulation and attachment to the Langendorff apparatus.

A custom-built Langendorff apparatus was used for whole porcine heart preparation.^8^ The apparatus consisted of a 5 L solution reservoir (custom supply from Radnoti Ltd.), an oxygen supply to oxygenate the physiological solution to maximal saturation, a heating coil to maintain optimal temperature of 37 ± 0.5°C (custom supply from Radnoti Ltd.) of the physiological solution, a bubble trap to remove air pockets from the system (custom supply from Radnoti Ltd.) and a high flow peristaltic pump to circulate solution around the system at a constant rate (Cole Parmer, UK). The physiological solution used was oxygenated Tyrode’s solution (10–3 moL/L: NaCl, 130; KCl, 4.05; MgCl_2_, 1.0; NaHCO_3_, 20; NaH_2_PO_4_, 1.0; glucose, 5.5; and CaCl_2_, 1.35; pH = 7.4). The solution chambers were connected to an aortic cannula, and oxygenated Tyrode’s solution was retrogradely perfused into the coronary arteries, thereby maintaining the metabolic, electrical, and contractile activity of the porcine hearts. The flow rate of the Langendorff pump was set to 75 mL/15 s. The perfusate exited the coronary circulation via the coronary sinus into the right atrium and then exited the right ventricle out of the severed pulmonary artery. A single-lead electrocardiogram (ECG) was recorded in the left ventricles of the hearts and recorded on LabChart (AD-Instruments, Oxford, UK). One electrode was also placed on the basal region of the left ventricle and connected to a MicroPace stimulator (CA, US) for pacing. We paced the left ventricle at 10% above the threshold of activation (usually 2 mV) and monitored for 20–30 min to ensure full stabilization and reversal of the ST segment elevation prior to starting the HFS protocol. If the hearts developed ventricular fibrillation, external DC cardioversion was performed to revert to sinus rhythm (SR).

**Figure 1:**
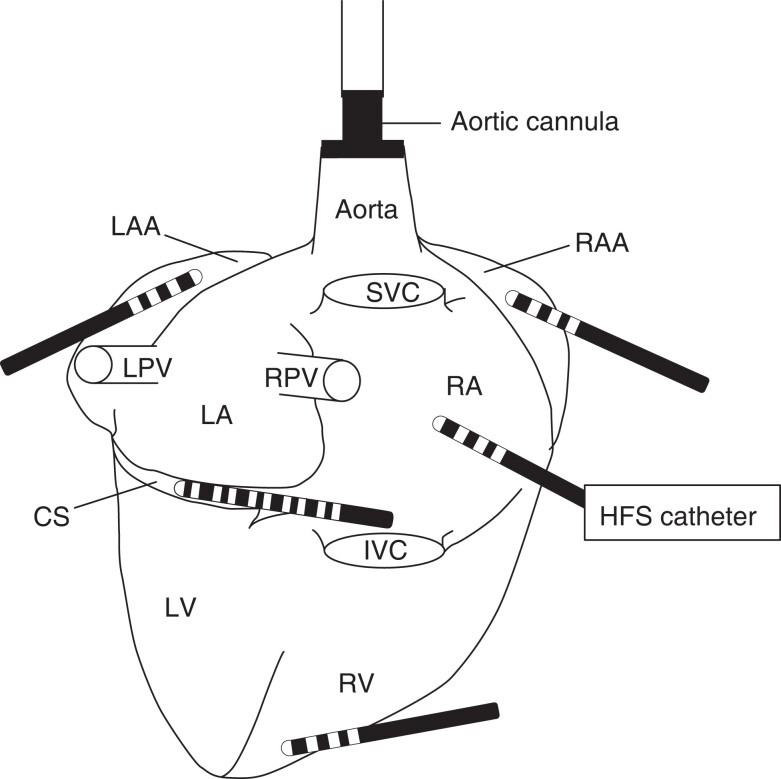
Catheter placements on the langendorff-perfused porcine heart. All catheters were fixed in a place using clamps to hold them into fixed position, and all the electrodes on the catheters were in direct contact with tissue. Three quadripolar catheters were positioned as follows: (i) directly beneath the left atrial appendage and over the left atrial surface, (ii) directly beneath the right atrial appendage and over the right atrial surface, (iii) directly beneath the right ventricular surface. One decapolar catheter was inserted into the coronary sinus via the superior vena cava. A 3.5 mm tip ablation catheter was used to deliver HFS from the distal two poles (labelled ‘HFS catheter’). This catheter was moved around the epicardium of the left and right atria for test with HFS. CS = coronary sinus; HFS = high frequency stimulation; IVC = inferior vena cava; LAA = left atrial appendage; LPV = left pulmonary vein; LV = left ventricle; RA = right atrium; RAA = right atrial appendage; RPV = right pulmonary vein; RV = right ventricle.

A Decapolar catheter was inserted into the coronary sinus via the superior vena cava (SVC). Three quadripolar catheters were placed at the right ventricular (RV) apex, and the left and right atrial appendages, and fixed into stable positions with clamps. A roving 3.5 mm tip ablation catheter was used to deliver HFS at multiple sites on the atrial epicardial surface (*Figure 1*). All intracardiac electrograms were recorded at 1000 Hz by an electrophysiology recording system (Bard EP, Lowell, MA). The ablation catheter was connected to a custom-built neural stimulator (V1.0, Tau-20) for delivery of HFS.

### Neural stimulator

In this study, we used Tau-20, a custom-built neural stimulator that replicates the functions of the Grass Stimulator (Astro-Med), the latter of which has been used commonly for basic and clinical autonomic studies previously. This grass stimulator can no longer be purchased or maintained in countries adopting the IEC/EN 60601-1 3^rd^ Edition Regulatory Standards (e.g. the European Union).

### Intrinsic cardiac nerve stimulation protocol

The left and right atria, including the superior vena cava and atrial appendages, were mapped with synchronized HFS. First, the atria were paced at high output (10–12 V) at a fixed rate (550–700 ms) above the intrinsic rate from the distal two electrodes of the HFS catheter. This was to ensure that there was adequate atrial capture and no ventricular capture. A short burst of HFS (80 ms, 20 Hz, 10–12 V) was then synchronized to each paced stimulus, for up to 15 trains or until an atrial ectopy or arrhythmia occurred. We aimed to test the whole surface of the atria, the superior vena cava, and appendages, with approximately 15–20 mm spacing between each test site. HFS was delivered on the epicardial surface of the atria only. A site was considered an ectopy-triggering (ET) site if there was a positive response to HFS, causing atrial ectopy, atrial tachycardia, or AF. A site was considered non-ectopy-triggering (non-ET) if none of these arrhythmic responses were elicited. All ET responses were re-tested up to four times to ensure reproducibility. If AF was triggered at any time, we waited for it to cardiovert to sinus rhythm before testing with HFS again. All AF cardioverted spontaneously, during the HFS protocol. Intracardiac recordings for the ET and non-ET sites were labelled on the Bard system, including descriptions of their anatomical locations.

### Immunohistochemistry of HFS-tested sites

One of the most reliable markers for cholinergic function is choline acetyltransferase (ChAT), a biosynthetic enzyme that is responsible for the synthesis of acetylcholine.^9^ We used this to quantify parasympathetic nerves in our porcine hearts. Tyrosine hydroxylase (TH) catalyses the initial and rate-limiting step in the biosynthetic pathway of catecholamines, including noradrenaline and adrenaline, within the postganglionic nerve terminals.^10^ Therefore, TH immunoreactivity (IR) is commonly used as a marker of sympathetic innervation, and we used this to identify sympathetic nerves in our porcine hearts.

At the end of the HFS protocol, all labelled ET and non-ET HFS tested sites were dissected down in a perpendicular plane to the atrial wall, aiming to include all layers of the atria (fat pad, epicardium, myocardium, endocardium) in each tissue. Each tissue block measured approximately 1.5 × 1.5 cm in size. Tissue samples were fixed using Tyrode’s solution-buffered formalin solution for 24 h, then transferred to phosphate buffered neutral solution (PBS). The tissues were further cut down to approximately 0.5 cm × 0.5 cm in size, and automatically processed using increasing concentrations of alcohol. The samples were cleared in xylene and then embedded in paraffin blocks. Multiple sister sections from each sample were taken that were 4–6 µm thick. One section from each tissue block was stained with Masson’s trichrome, and the remaining sections were used for immunohistochemistry with chicken polyclonal antibody to tyrosine hydroxylase (TH) (Abcam ab76442), and rabbit monoclonal antibody to choline acetyltransferase (ChAT) (Abcam ab178850), in separate sections. After washing, secondary antibodies conjugated with fluorescent markers were applied. A goat anti-chicken Alexa-Fluor 568 (Abcam ab175477) for anti-TH primary antibody and a donkey anti-rabbit Alexa-Fluor 488 (Abcam ab150073) for anti-ChAT primary antibody were used.

TH antigen retrieval was performed by boiling in citrate buffer (pH 6.0) for 10 min, and for ChAT, at pH 6 and pH 9, in citrate and tris-EDTA buffer, respectively. This determined that pH 9 tris-EDTA is the superior solution for ChAT antigen retrieval. Antigen-blocking was performed for one hour with 5% normal goat and donkey serum in PBS. Antibody dilutions were then prepared in blocking serum to reduce non-specific binding. Primary antibodies were titrated to ensure optimal dilution (1:250) and allowed to react for 1 h at room temperature, then washed three times in PBS with 0.1% Triton X-100. Secondary antibodies were used at a dilution of 1:500, and left overnight at 5°C. Following a further three washes in PBS-Triton X, coverslips were added, using a DAPI (4′,6-diamidino-2-phenylindole)-positive mounting agent.

A no-primary PBS solution control was performed for all samples to exclude false positive signals caused by non-specific binding of the secondary antigen to tissue. A rabbit IgG isotype control for monoclonal anti-ChAT was performed to exclude non-specific Fc receptor binding or other cellular protein interactions with the primary antibody to tissue. Any non-specific fluorescence identified in the controls and in the reactive samples was excluded from the analysis. An IgG isotype control was not an appropriate test for the anti-TH antibody, due to its polyclonal nature. Instead, we performed a no-primary antibody control test for anti-TH antibodies. Positive control tests were performed with a sympathetic nerve trunk and vagus nerves from one of the pigs, known to express TH and ChAT, respectively, to validate our immunohistochemistry technique (see Supplementary material online, *Figure S1*).

### Image acquisition and analysis

Brightfield and fluorescence imaging of the entire tissue area was performed using a 10 × objective on a Zeiss AxioObserver microscope controlled by ZEN Pro software (Zeiss). Areas of interest were imaged further using 20 × and 40 × objectives. Image analysis was performed using ImageJ (FIJI) software. Tissue anatomy was first examined from brightfield images. The epicardial, myocardial, and connective tissue layers of each sample were determined by manual selection of the structures by anatomy, and their area was measured automatically using the software. The colour deconvolution function helped to differentiate connective tissue from the myocardium automatically.

Nerves with immunofluorescent or IR signals were identified manually, by examining the microscopic images. Masson’s Trichrome-stained samples were used as references to identify important landmarks and structures, as well as to validate all IR nerves identified. Ganglia were clearly distinguishable by their epineurium, encapsulated bundles of neurones that were densely packed and IR. This helped to differentiate very small ganglia from false-positive IR signals. Every IR-stained sample had a control to compare against, to further reduce the probability of analysing false positive signals.

For the quantification of nerves, we adjusted for the variation in the sampled tissue sizes by calculating the number of nerves per cm^2^ of tissue, expressed as ‘nerve density’. This was calculated by dividing the number of nerves identified by the sampled tissue surface area examined. The cross-sectional areas (CSA) of nerves were automatically calculated by the ZEN Pro software by drawing around each nerve structure.

The researcher performing microscopic examinations and nerve quantification was blinded to the tissue’s functional response to HFS (ET or non-ET).

### Statistical analysis

GraphPad 5 (Prism, San Diego, California) was used for all statistical testing. Comparisons of means were performed using unpaired *t*-tests. Based on previous studies, GPs have the densest clusters of neurones in the intrinsic cardiac autonomic nervous system,^11^ and their direct stimulation can lead to atrial arrhythmias. Therefore, we tested the hypothesis that HFS at a random site that reproducibly triggers atrial arrhythmia has a higher density of ganglia than a non-ET site using a one-tailed *t*-test, testing for a statistical difference in this direction only. All other comparisons of means used two-tailed *t*-tests. A two-way ANOVA was also performed for multiple comparisons. Continuous variables were expressed as mean ± standard deviation. The threshold for statistical significance was *P* < 0.05.

## Results

**Figure 2:**
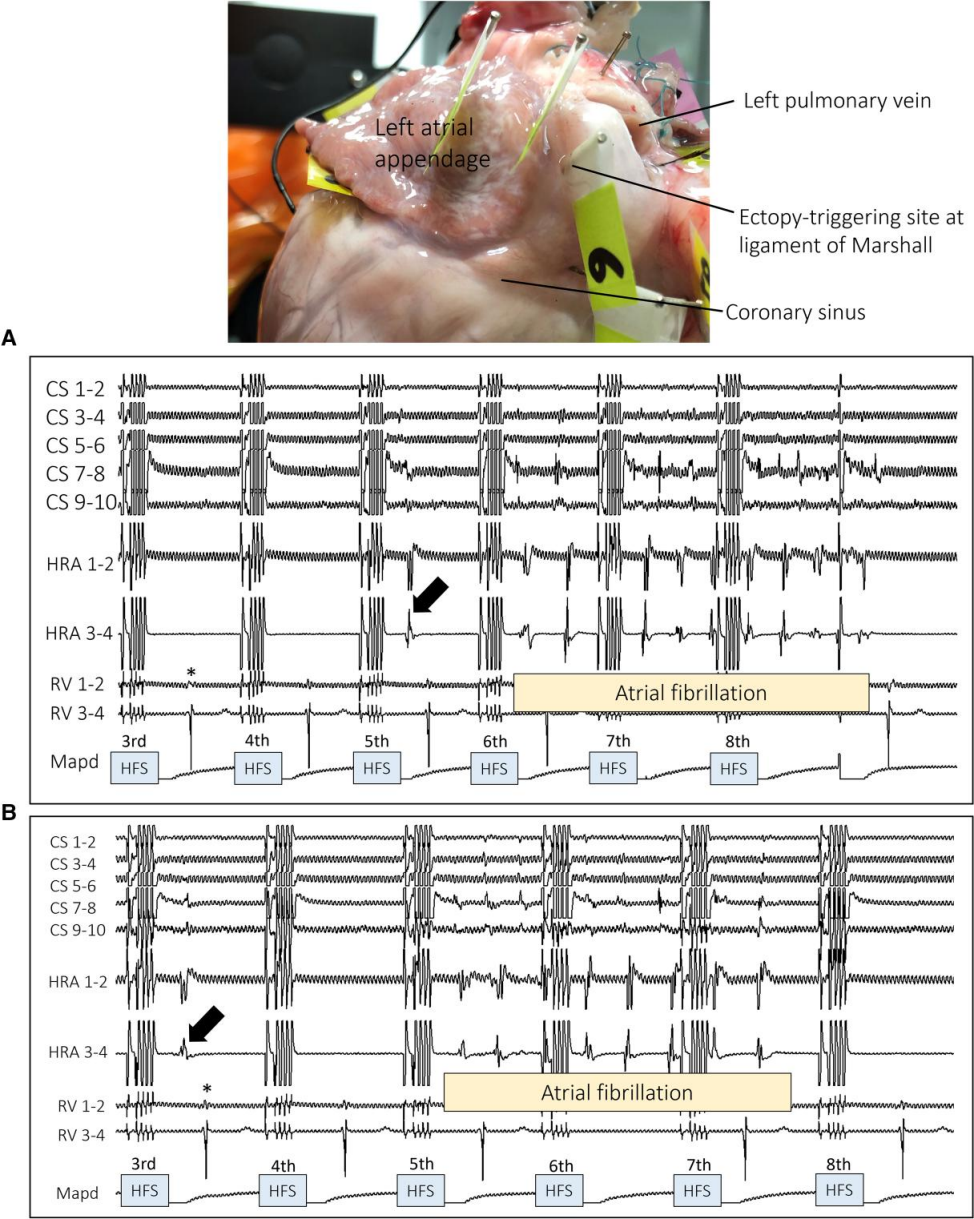
Example of HFS-tested site triggering reproducible atrial ectopy and atrial fibrillation. The top photograph shows the local site of HFS at a position labelled ‘6’, immediately lateral to the base of the left atrial appendage (the ligament of Marshall) in the left atrial posterior wall. The same site was tested up to four times, which reproducibly triggered atrial fibrillation in all instances. Trace A shows an atrial ectopy triggered with the earliest activation in the coronary sinus (arrow) after the fifth HFS train, with a coupling interval of 276 ms from the pacing stimulus. After the sixth HFS, a short run of atrial fibrillation was triggered, which self-terminated. Trace B shows a repeat HFS test at the same anatomical site as tested in A. After the third HFS, an atrial ectopy (arrow) was triggered with a coupling interval of 2 ms from the pacing stimulus. After the fifth HFS, atrial fibrillation was triggered with the earliest activation from CS 7–8/9–10. *=ventricular conduction from paced atrium; HFS = high frequency stimulation; HRA = high right atrium; Mapd = mapping catheter distal; RV = right ventricle; V = ventricular.

**Figure 3:**
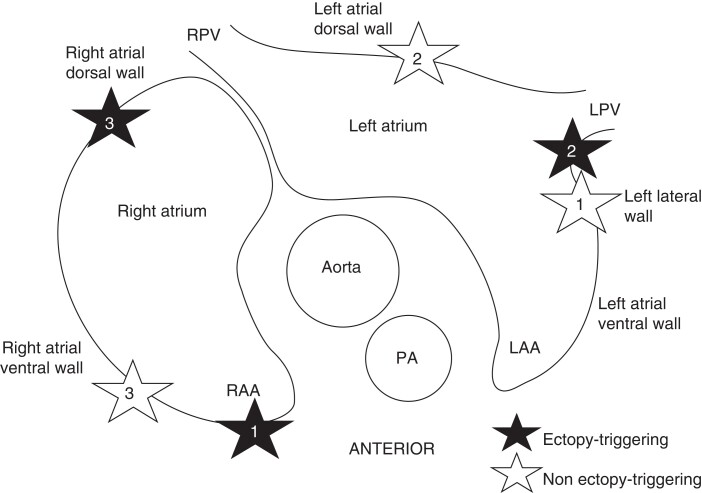
Anatomical sites of sampled ectopy and non-ectopy-triggering sites. A diagram showing a transverse section of a porcine heart. Black stars represent ectopy-triggering sites sampled for analysis. White stars represent non-ectopy-triggering sites sampled for analysis. Four ectopy-triggering sites were from the right atrium (three dorsal right atrium, one right atrial appendage) and two from the left atrium (two ridges between the left atrial appendage and the left pulmonary vein; ligament of Marshall). Three non-ectopy-triggering sites were from the right atrium (three ventral right atrium), and three were from the left atrium (two dorsal left atrium, one left lateral wall of left atrium). LPV = left pulmonary vein, PA = pulmonary artery, RPV = right pulmonary vein.

**Figure 4:**
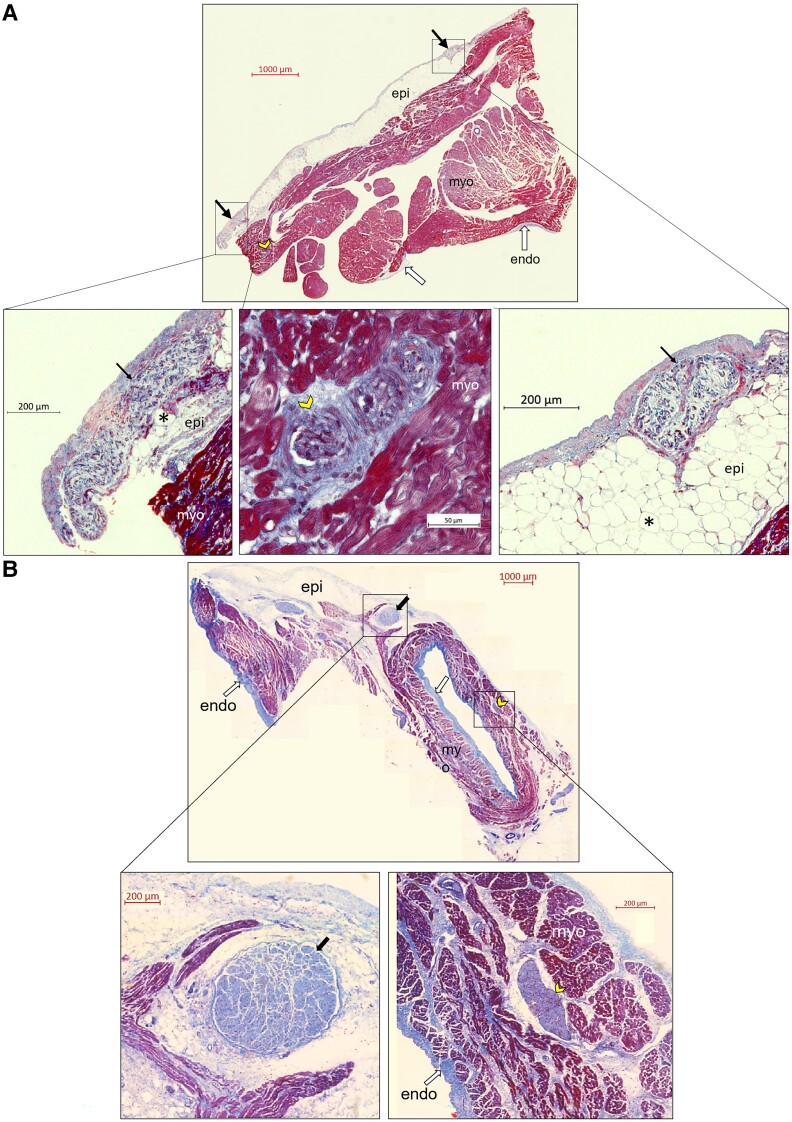
Example of masson’s trichrome-stained epicardial and myocardial ganglia at high frequency stimulation-tested sites. These were cross-sectional sections stained with Masson’s Trichrome at sites that triggered reproducible atrial ectopy with high frequency stimulation (*A*) and no atrial arrhythmias with high frequency stimulation (*B*). The Masson’s Trichrome stain allowed clear differentiation of nerve tissue from other tissue structures. All nerves (arrows and arrowheads) were stained light to dark purple, always surrounded by dense, light blue connective tissue that represented the epineurium. Muscle fibres were stained red, and collagen in the extracellular matrix was stained blue. The epicardial layer (epi) was abundant in adipocytes (*), and the ganglia in this layer were often the largest nerve structure in the entire tissue section (black arrows). In both (*A*) and (*B*), the top photographs show the whole sections in view. The bottom rows show magnified views of the boxed ganglia from the top photographs. (*A*) Anatomically, this site was just posterior to the base of the right atrial appendage. The bottom left shows a long-axis view of an epicardial ganglia (1013 µm × 147 µm), the bottom right shows a short-axis view of another epicardial ganglia (301 µm × 179 µm). In the middle of the bottom row, a short-axis view of smaller myocardial ganglia is seen (yellow arrowhead; 195 µm × 101 µm). (*B*) Anatomically, this site was in the left lateral wall of the left atrium. The bottom left shows an epicardial ganglia (689 × 623 µm). The bottom right shows a smaller myocardial ganglia (yellow arrowhead; 451 × 157 µm). Endo = endocardium; epi = epicardium; HFS = high frequency stimulation; myo = myocardium.

Four porcine hearts had a total of 63 atrial sites tested with HFS across all hearts, identifying six (10%) ET sites (*Figure 2*). No ventricular arrhythmias occurred during the HFS protocol. Four ET sites were from the right atrium (three dorsal right atrium and one right atrial appendage) and two from the left atrium (two ridges between the left atrial appendage and the left PV; ligament of Marshall). Six non-ET sites were randomly sampled arbitrarily from the hearts, from both atria (three ventral right atria, two dorsal left atrium, one left lateral wall of left atrium) (*Figure 3*). The breakdown of tissues sampled from each porcine heart is shown in *Table 1*. The proportion of epicardium, myocardium, and endocardium were similar across all ET tissues. However, in two of the six non-ET tissues, there was a disproportionately larger proportion of the epicardium than the other layers, and one non-ET tissue had no epicardium. The epicardium contained connective tissue, ganglia, vessels, and fat. Connective tissue penetrated the myocardium and was often continuous with the epicardium. Ganglia, and thin nerve fibrils were also present in the myocardium (*Figure 4*). We identified 678 IR nerves, of which 429 (63%) were ChAT-IR, which stains for parasympathetic nerves, and 249 (37%) were TH-IR, which stains for sympathetic nerves.

**Table 1:** Breakdown of tissues sampled from all porcine hearts

Porcine hearts	Sampled tissues
1	4 non-ET (3 LA, 1 RA) 4 ET (1 LA, 3 RA)
2	1 non-ET (RA)
3	1 ET (RA)
4	1 non-ET (RA) 1 ET (LA)

ET = ectopy-triggering; LA = left atrial; RA = right atrial.

### Larger sympathetic nerves compared to parasympathetic nerves in both ET and non-ET sites

**Figure 5:**
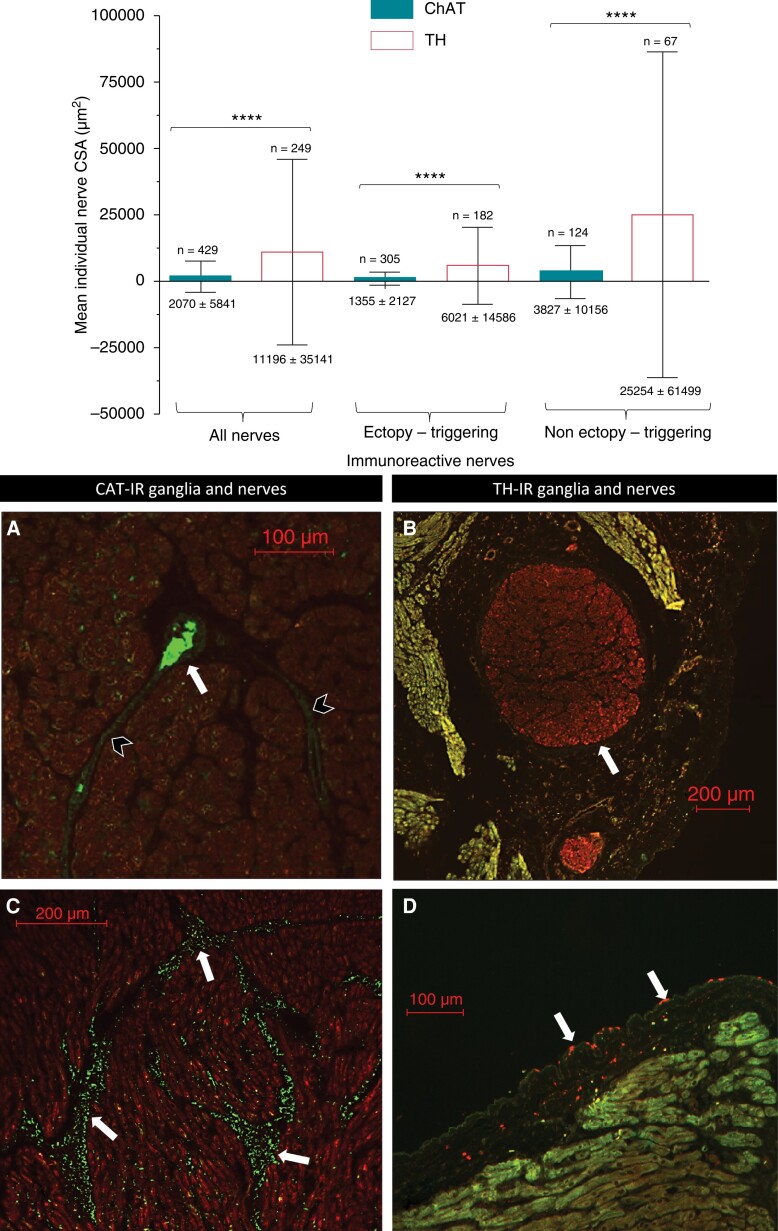
Comparison of the mean cross-sectional area of individual choline acetyltransferase immunoreactive vs. tyrosine hydroxylase immunoreactive nerves. The bars from the bar graph represent the mean, and the error bars represent the standard deviation of the mean (the precise values are at the bottom of the error bars). The results showed that the mean cross-sectional area of tyrosine hydroxylase immunoreactive nerves (*n* = 249) was significantly larger than choline acetyltransferase immunoreactive nerves (*n* = 429) (µm^2^: 11 196 ± 35 141 vs. 2070 ± 5841; *P* < 0.0001). Likewise, this pattern was also observed within ET (μm^2^: 6021 ± 14 586 tyrosine hydroxylase immunoreactive vs. 1355 ± 2127 choline acetyltransferase immunoreactive; *P* < 0.0001) and non-ET sites (μm^2^: 25 254 ± 61 499 tyrosine hydroxylase immunoreactive vs. 3827 ± 10 156 ChAT-IR; *P* < 0.0001). The photographs below the graph show typical examples of choline acetyltransferase immunoreactive and tyrosine hydroxylase immunoreactive nerves in different ranges of sizes from cross-sections. (*A*) Myocardial choline acetyltransferase immunoreactive ganglia (white arrow; 3948 µm^2^) with longitudinal view of the branching out axons (black arrowheads); (*B*) epicardial tyrosine hydroxylase immunoreactive ganglia (white arrow; 337 718 µm^2^); (*C*) myocardial choline acetyltransferase immunoreactive nerves (white arrow; <200 µm^2^) in dense, compact formation, (*D*) epicardial tyrosine hydroxylase immunoreactive nerves (white arrow; <50 µm^2^) scattered throughout the epicardium, which was an uncommon finding. The scale bars are shown as size references.

Within both ET and non-ET sites, the CSA of individual TH-IR nerves was significantly larger than that of ChAT-IR nerves (μm^2^: 11 196 ± 35 141 vs. 2070 ± 5841; *P* < 0.0001) (*Table 2* and *Figure 5*). Likewise, this pattern was also observed within ET sites (μm^2^: 6021 ± 14 586 TH-IR vs. 1355 ±2127 ChAT-IR; *P* < 0.0001) and non-ET sites (μm^2^: 25 254 ± 61 499 TH-IR vs. 3827 ±10 156 ChAT-IR; *P* < 0.0001).

**Table 2:** Comparison between ChAT-IR and TH-IR nerves

	ChAT-IR nerves (*n* = 429)	TH-IR nerves (*n* = 249)	*P* Value
Density of nerves (no. of nerves/cm^2^)	68.0 ± 68.2	48.0 ± 46.0	0.62
ȃET sites (no. of nerves/cm^2^)	92.4 ± 79.4	69.9 ± 54.1	0.82
ȃNon-ET sites (no. of nerves/cm^2^)	43.6 ± 49.9	26.1 ± 24.1	0.57
CSA of individual nerves per tissue (µm^2^)	2070 ± 5841	11 196 ± 35 141	<0.0001
ȃET sites (µm^2^)	1355 ± 2127	6021 ± 14 586	<0.0001
ȃNon-ET sites (µm^2^)	3827 ± 10 156	25 254 ± 61 499	<0.0001

Values are presented as mean ± SD.

Six hundred seventy-eight IR nerves were examined from four porcine hearts. The CSA of individual nerves per tissue was significantly larger with TH-IR nerves compared to ChAT-IR nerves (µm^2^: 11 196 ± 35 141 vs. 2070 ± 5841; *P* < 0.0001). Similar pattern was observed within ET and non-ET sites. There was a trend towards increased density of ChAT-IR nerves compared to TH-IR nerves (nerves/cm^2^: 68.0 ± 68.2 vs. 48.0 ± 46.0; *P* = 0.62), but this was not statistically significant. Similar pattern was observed within ET and non-ET sites.

ChAT-IR = choline acetyltransferase immunoreactive; CSA = cross sectional area; ET = ectopy-triggering; IR = immunoreactive; non-ET = non-ectopy-triggering; TH-IR = tyrosine hydroxylase immunoreactive.

Typical examples of ChAT-IR and TH-IR nerves are shown in *Figure 5*. We often found ChAT-IR ganglia in the myocardium (*Figure 5A*), and some ChAT-IR nerves were smaller and in more compact formation in the myocardium (*Figure 5C*). TH-IR ganglia were often found in the epicardium (*Figure 5B*) and some were scattered across the epicardium, which was an uncommon finding (*Figure 5D*).

**Figure 6:**
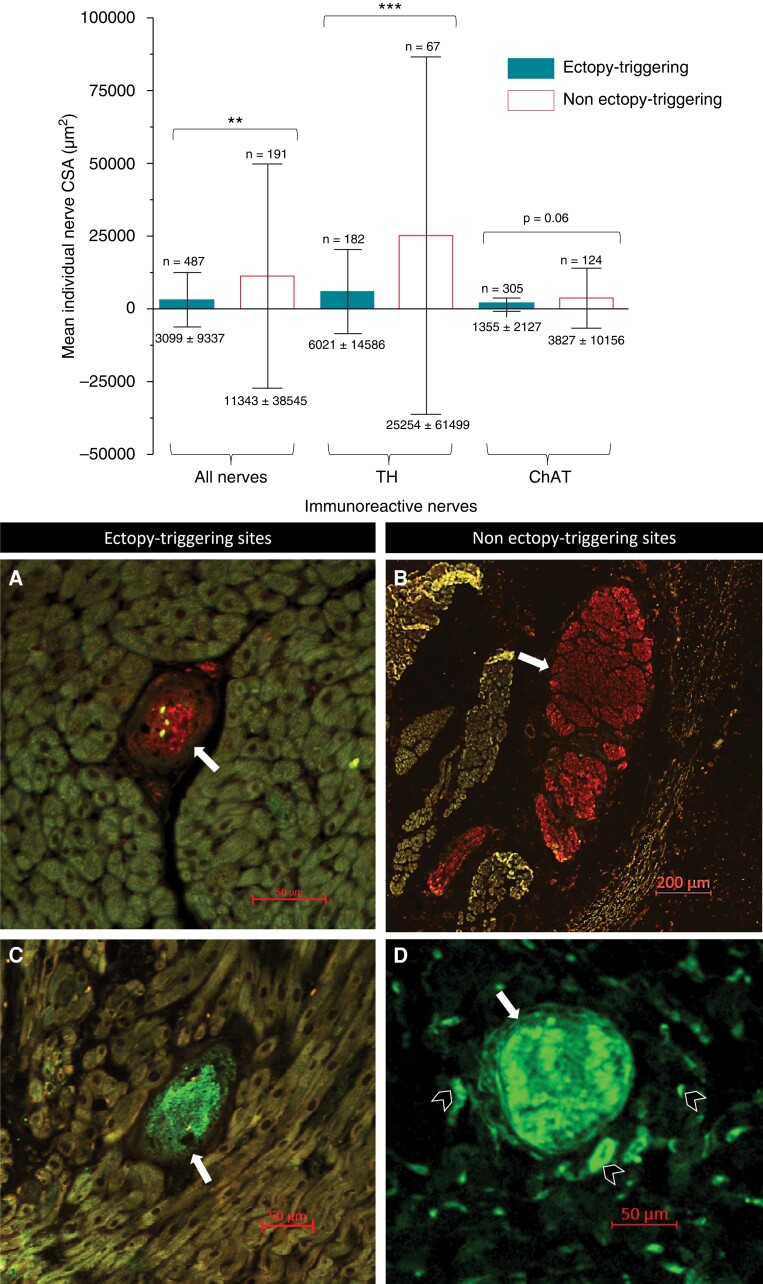
Comparison of the mean cross-sectional area of nerves in ectopy-triggering and non-ectopy-triggering sites from cross-sections of porcine tissues. Six hundred seventy-eight immunoreactive nerves were examined from six ectopy-triggering (*n* = 487 nerves) and six non-ectopy-triggering (*n* = 191 nerves) sites from four porcine hearts. The bars from the bar graph represent the mean and the error bars represent the standard deviation of the mean. The precise values are on the bottom of the error bars. The total *n* numbers analysed are at the top of the error bars. The results showed that the cross-sectional area of nerves in ectopy-triggering was significantly smaller than in non-ectopy-triggering sites (µm^2^: 3099 ± 9337 vs. 11 343 ± 38 545; *P* = 0.001). The biggest significant difference in size was seen in tyrosine hydroxylase immunoreactive nerves of non-ectopy-triggering (*n* = 182; 25 254 ± 61 499 µm^2^) vs. ectopy-triggering sites (*n* = 67; 6021 ± 14 586 µm^2^; *P* = 0.0006). There was also a trend for larger choline acetyltransferase immunoreactive nerves in non-ectopy-triggering (*n* = 124; 3827 ± 10 156 µm^2^) compared to ectopy-triggering sites (*n* = 305; 1355 ± 2127 µm^2^), but this was not statistically significant (*P* = 0.06). The photographs below the graph show typical examples of ectopy-triggering and non-ectopy-triggering ganglia identified from cross-sections. (*A*) myocardial tyrosine hydroxylase immunoreactive ganglia in an ectopy-triggering site (white arrow; 3806 µm^2^), (*B*) epicardial tyrosine hydroxylase immunoreactive ganglia in a non-ectopy-triggering site (white arrow; 278 703 µm^2^), (*C*) myocardial choline acetyltransferase immunoreactive ganglia in an ectopy-triggering site (white arrow; 6955 µm^2^), (*D*) epicardial choline acetyltransferase immunoreactive ganglia (white arrow; 9948 µm^2^) in a non-ectopy-triggering site, which was also surrounded by smaller choline acetyltransferase immunoreactive nerves (black arrowhead). The scale bars are shown as size references.

Although TH-IR nerves had a significantly larger CSA than ChAT-IR nerves overall, TH-IR nerves had a significantly smaller CSA in ET compared to non-ET sites (μm^2^: 6021 ± 14 586 vs. 25 254 ± 61 499; *P* < 0.001). There was also a trend to smaller CSA for ChAT-IR nerves at ET sites, though this did not reach statistical significance (μm^2^: 1355 ± 2127 vs. 3827 ± 10 156; *P* = 0.06). Overall, IR nerves in ET sites were significantly smaller than in non-ET sites (μm^2^: 3099 ± 9337 vs. 11 343 ± 38 545; *P* = 0.001) (*Figure 6* and *Table 3*).

**Table 3:** Comparison between ET and non-ET sites

Measure	ET sites (*n* = 6)	Non-ET sites (*n* = 6)	*P* Value
Density of nerves (no. of nerves/cm^2^)	162.3 ± 110.9	69.7 ± 72.5	0.047
ȃTH-IR nerves (no. of nerves/cm^2^)	69.9 ± 54.1	26.1 ± 24.1	0.12
ȃChAT-IR nerves (no. of nerves/cm^2^)	92.4 ± 79.4	43.6 ± 49.9	0.07
CSA of individual nerves per tissue (µm^2^)	3099 ± 9337	11 343 ± 38 545	0.001
ȃTH-IR nerves (µm^2^)	6021 ± 14 586	25 254 ± 61 499	<0.001
ȃChAT-IR nerves (µm^2^)	1355 ± 2127	3827 ± 10 156	0.06

Values are presented as mean ± SD.

Six ET and six non-ET sites were examined on four porcine hearts. There was a significantly increased density of nerves in ET compared to non-ET sites (nerves/cm^2^:162.3 ± 110.9 vs. 69.7 ± 72.5; *P* = 0.047). A similar trend was observed within TH-IR nerves and within ChAT-IR nerves, but this was not statistically significant. The CSA of individual nerves in ET was significantly larger than in non-ET sites (µm^2^: 11 343 ±38 545 vs. 3099 ±9337; *P* = 0.001). The greatest difference of CSA between ET and non-ET sites was seen within TH-IR nerves (µm^2^: 25 254 ±61 499 vs. 6021 ±14 586; *P* < 0.001).

Abbreviations are the same as in *Table 2*.

### Increased density of nerves in ET compared to non-ET sites

Out of the total 678 immunoreactive nerves identified, 487 (72%) were from ET sites and 191 (28%) were from non-ET sites. There were similar proportions of TH-IR and ChAT-IR nerves at ET and non-ET sites. For TH-IR nerves, there were 182 (37%) vs. 67 (35%) in ET and non-ET sites, respectively. For ChAT-IR nerves, there were 305 (67%) vs. 124 (65%) at ET and non-ET sites, respectively (*P* = 0.60).

**Figure 7:**
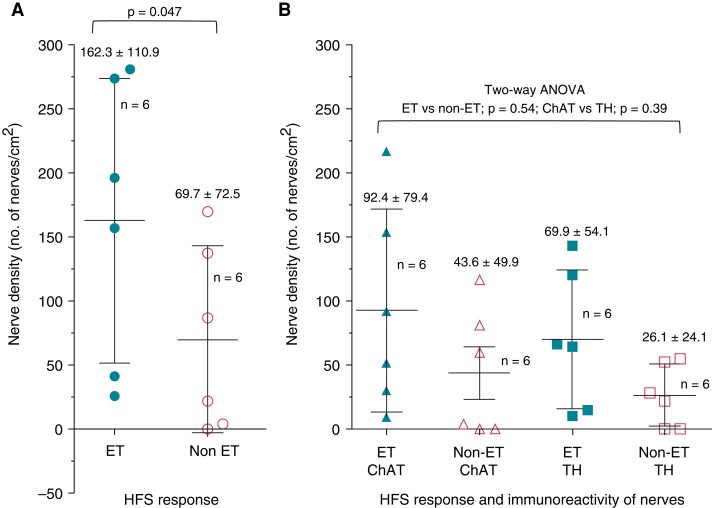
Nerve density comparison between ectopy-triggering vs. non-ectopy-triggering and choline acetyltransferase vs. tyrosine hydroxylase. The graph illustrates the mean and the standard deviation as error bars. The precise values are displayed on top of the error bars, and the total *n* numbers are beside each scatter graph. Six hundred seventy-eight immunoreactive-nerves were examined from six ectopy-triggering and six non-ectopy-triggering sites on four porcine hearts. The nerve density of a tissue was calculated as the average number of nerves per cm^2^ of the porcine tissue examined. Two different statistical analyses were performed for comparison between the independent variables: ectopy-triggering vs. non-ectopy-triggering, and choline acetyltransferase vs. tyrosine hydroxylase, and the dependent variable, the average nerve density. In (*A*), a one-tailed *t*-test was performed. There was a significantly increased denstiy of nerves in ectopy-triggering compared to non-ectopy-triggering sites (nerves/cm^2^: 162.3 ± 110.9 vs. 69.7 ± 72.5; *P* = 0.047). In (*B*), a two-way analysis of variance was performed. This showed that there was a trend towards significance when comparing ectopy-triggeringvs. non-ectopy-triggering [f(1,20) = 4.19; *P* = 0.054], but no significance when comparing choline acetyltransferase vs. tyrosine hydroxylase [f(1,20) = 0.78; *P* = 0.39] on their effects on nerve density.

Overall, comparison between the means showed that there was a significantly increased nerve density in ET compared to non-ET sites (nerves/cm^2^: 162.3 ± 110.9 vs. 69.65 ± 72.48; *P* = 0.047) (*Figure 7A*). Specific comparisons between ChAT-IR and TH-IR nerves within ET and non-ET sites showed that there was a trend towards increased nerve density in ChAT-IR in ET compared to non-ET sites (nerves/cm^2^: 92.4 ± 79.4 vs. 43.6 ± 49.9; *P* = 0.07) and similarly in TH-IR nerves (nerves/cm^2^: 69.9 ± 54.1 vs. 26.1 ± 24.1; *P* = 0.12) (*Table 3*). A two-way ANOVA was also performed for multiple comparisons. This showed that there was a trend towards significance when comparing ET vs. non-ET [f(1,20) = 4.19; *P* = 0.054], but no significance when comparing ChAT vs. TH [f(1,20) = 0.78; *P* = 0.39] on their effects on nerve density (*Figure 7B*). There was also no significant interaction between HFS response type and the immunoreactivity type of nerves [(F(1,20) = 0.01, *P* = 0.91].

## Discussion

This is the first study to characterize the differing topological and immunohistochemical properties of the intrinsic cardiac autonomic nervous system of a porcine heart, based on two different neurotransmitters (ChAT and TH), and correlate these findings to the local electrophysiological response to neural stimulation. Our main findings were: (i) there was a significantly increased density of nerves in ET compared to non-ET sites, (ii) overall, TH-IR nerves, which represent sympathetic nerves, were significantly larger than ChAT-IR nerves, which represent parasympathetic nerves. However, (iii) in ET sites, TH-IR nerves were significantly smaller than in non-ET sites.

### Neurotransmitters in the ganglia

The intrinsic cardiac ganglia contain a heterogeneous population of neurones, regarding their morphology, electrophysiology, as well as their associated neurotransmitters and phenotypes. This heterogeneity of neurones may correlate to different local controls over cardiac function. In one detailed anatomical study of the porcine intrinsic cardiac autonomic nervous system, there were approximately 82 ganglia of various sizes found per pig heart, containing on average 1600 neurones.^12^ The number of neurones per ganglia ranged from only one or two to more than 100 neurones. The largest collection of neurones was on the ventral surface of the right atrium. As in our study, the authors found ganglia embedded in the epicardium as well as in the deeper underling cardiac muscle fascicles. The porcine neuronal organization is similar to that found in human intrinsic cardiac ganglia.^13^

A gross histochemical staining for parasympathetic nerves in whole porcine hearts has been studied by Ulphani *et al.*^14^ This showed that parasympathetic nerves were densest in the endocardium than in the epicardium. However, the diameter of parasympathetic nerves was the thickest in the epicardium than in the endocardium. In our study, we also found a higher density of parasympathetic nerves within the myocardium, than the epicardium.

In another porcine heart study by Crick *et al,*^15^ the atrial GPs contained both parasympathetic and sympathetic cell bodies, as we have found in our study. Parasympathetic nerves (stained for AChE-IR) were the densest subtype of nerves stained in porcine hearts, followed by sympathetic nerves (stained for TH-IR). This is also consistent with our findings.

### Smaller and denser nerves in ectopy-triggering sites compared to non-ectopy-triggering sites

We found that ET sites contained significantly smaller-sized nerves that were denser than in non-ET sites. As in the study by Ulphani *et al,*^14^ the largest nerves were usually found in the epicardium of the atria, and the smallest in the myocardium or the endocardium. Therefore, the smaller size and higher density of nerves in ET sites compared to non-ET sites indicate a unique functional and anatomical characteristic of the ectopy-triggering ability. One possible explanation of this appearance is that small and densely packed nerves occupy a greater surface area of a region in the tissue, which allows for greater density of interconnecting nerves between ganglia that are responsible for triggering atrial arrhythmias.

A higher concentration of neurotransmitters may be released from a dense network of nerve axons occupying a larger surface area, as the communicating ganglia are stimulated. It is possible that the culprit ganglia that causes atrial arrhythmias is not at the local site of HFS, but at a more distant site, communicating with the local interconnecting nerves. This may explain the significantly larger ganglia present at non-ET sites compared to ET sites. Crick *et al*^15^ also found that there was a contrasting pattern of distribution of GPs in the epicardium compared to the endocardium, suggesting that the epicardial GPs can function independently of the endocardial nerves. This hypothesis is supported by the functional and histological correlations we have demonstrated in our study. We identified that the smaller and denser network of nerves, the majority of which are in the myocardium of the atria, are responsible for the ET effect. This suggests that the larger epicardial GP may function independently of the smaller network of intrinsic cardiac nerves in the myocardium and the endocardium.

### The mechanism for ectopy-triggering responses to nerve stimulation

Previously, stimulating the local innervation at the canine PVs had shown a disparity in the local tissue electrophysiological properties by shortening the local action potential from stimulation of the parasympathetic nerves and increasing the calcium transient from stimulation of the sympathetic nerves. The combination of the two together led to early post-depolarizations and arrhythmogenesis.^2^ This may be the underlying mechanism for the ET properties in our study, which stimulate both the parasympathetic and sympathetic intrinsic cardiac nerves. Hyperinnervation has also been demonstrated in canines with AF compared to canines with no AF by immunohistochemical methods.^16^ In another study, heterogeneous sympathetic denervation increased the dispersion of atrial refractoriness and created substrates for sustained AF.^17^ We compared the density of sympathetic and parasympathetic nerves within ET and non-ET sites, and these were not statistically significant.

### Clinical implications

Our findings have important clinical implications for the treatment of AF by targeting potential autonomic triggers. In some cases, HFS-guided ‘ectopy-triggering GP’ (ET-GP) ablation without any additional PVI has shown to be effective at preventing AF recurrence. Previously, a prospective, randomized, controlled trial (GANGLIA-AF) targeted specifically the ET-GPs without PVI, and at 12 months’ follow-up, 58% of patients were free of atrial arrhythmias, compared to 64% after PVI.^7^ The clinical ET-GP mapping protocol involves delivering HFS from the endocardium, which is presumed to stimulate epicardial GPs that are interconnected with smaller and more downstream nerves in the myocardium and the endocardium. However, from the findings of this study, HFS may be identifying local areas that have a smaller and higher density of nerves, both parasympathetic and sympathetic nerves. In this case, radiofrequency ablation of the local ET HFS site may destroy mostly the endocardial and myocardial nerves that may be interconnected with more distant ‘culprit’ epicardial ganglia. Incomplete destruction of the epicardial ganglia by only ablating the local nerve axons may encourage neural plasticity. This may explain why, several months after GP ablation, there is reversal of HRV parameters, as a marker of neuromodulation, and recurrence of atrial arrhythmias.^18^ It should also be noted that in the GANGLIA-AF trial, there was a significantly shorter radiofrequency ablation time with ET-GP ablation compared to PVI.^7^ This suggests that a wider cloud of ablation at local ET sites may be required to eliminate the more distant GPs, as well as to destroy the smaller, densest part of the intrinsic cardiac nerves to prevent long-term atrial arrhythmia recurrence.

Recent, large, randomized controlled trials in AF and PVI have shown a range of long-term successes between 53–75% at preventing atrial arrhythmia recurrence,^5,19,20^ comparable to GANGLIA-AF trial’s result with PVI. It may be possible that an adjunctive approach to AF with PVI and ET-GP ablation may yield superior clinical outcomes by eliminating PV and non-PV sources of AF.

Apart from HFS, radionucleotide imaging using the ^123^I-metaiodobenzylguanidine (MIBG)^21^ and single photon emission computed tomography imaging has been proposed as an alternative technique to visualize the atrial GPs.^22^ However, MIBG is a guanethidine analogue of norepinephrine that targets the norepinephrine transporter. This would selectively image the sympathetic nerves of the intrinsic cardiac nervous system. Our results further support the evidence in the literature, that there are abundant parasympathetic nerves in the atria, which may be missed using this technique.

### Limitations

The sample size is small, which may have impacted the results. Despite this, we found significant differences between the variables tested in this study, and the researcher performing microscopic examination and quantifying nerves was blinded to whether a sample was ET or non-ET. Two of the six non-ET tissues had disproportionately larger epicardium than the other layers, and one non-ET tissue had no epicardium. This may have affected the results when comparing them against ET tissues. The quantification of ganglia and nerves in this study likely underestimates the true density of nerves, due to the cross-sectional sampling of the HFS-tested sites. The minimum stabilization period for the hearts was 20 min, while the maximum was 30 min. The entire pacing protocol with HFS ranged between 30–45 min. The time it took to fix the sampled porcine tissues after ending the testing protocol ranged between 30–45 min between hearts. These variations in time may have impacted the concentration of neurotransmitters present at the time of fixing the tissue, and subsequently the density of nerves identified. This experiment used decentralized hearts that relied on local circuit neurones that functioned independently of stimulation. The lack of extrinsic cardiac ANS input may have had some effect on the atrial electrophysiology. There was a smaller proportion of ET sites (10%) identified in this study compared with the GANGLIA-AF clinical study, which identified 18.5% of ET sites with HFS. This may be reflective of the less dense HFS mapping performed in our study, the absence of the extrinsic cardiac ANS, and also the physiological difference in species.

In humans, there is substantial evidence regarding the influence of sex and age on the pathophysiology of AF.^23^ In this study, for consistency, we only used young, female porcine hearts. There may be important gender and age differences in the structure and function of the intrinsic cardiac ANS and the pathophysiology of AF that were not reflected in the results of our study and should form the basis of future studies.

Finally, our findings in this porcine heart study may not all translate to human hearts. Previous anatomical study of human hearts and their innervation found parasympathetic innervation to be significantly denser in the atria than in the ventricles,^24^ whilst a previous porcine heart study found significantly more dense innervation in the ventricles than in the atria.^14^ It is not clear whether this difference is species-related or due to methodologic differences.

## Conclusion

In decentralized porcine hearts, HFS can identify local sites that trigger atrial arrhythmias. These sites contain a smaller and higher density of nerves (both parasympathetic and sympathetic), compared to local sites that do not trigger atrial arrhythmias. The majority of these nerves were in the myocardium of the atria. This has potentially important clinical implications in devising an effective therapeutic strategy that targets the autonomic triggers of AF.

## Supplementary Material

euac176_Supplementary_Data

## Data Availability

The data that support the findings of this study are available from the corresponding author, upon reasonable request.
