# Supplementary material for: Immunohistochemical characteristics of local sites that trigger atrial arrhythmias in response to high-frequency stimulation
Source: Europace. 2022 Oct 19;25(2):726–38. doi: 10.1093/europace/euac176 (PMC9935019; doi:10.1093/europace/euac176)
Supplement: euac176_Supplementary_Data [file euac176_supplementary_data.zip › EP Europace-supplement.docx]

**Supplementary Figures**


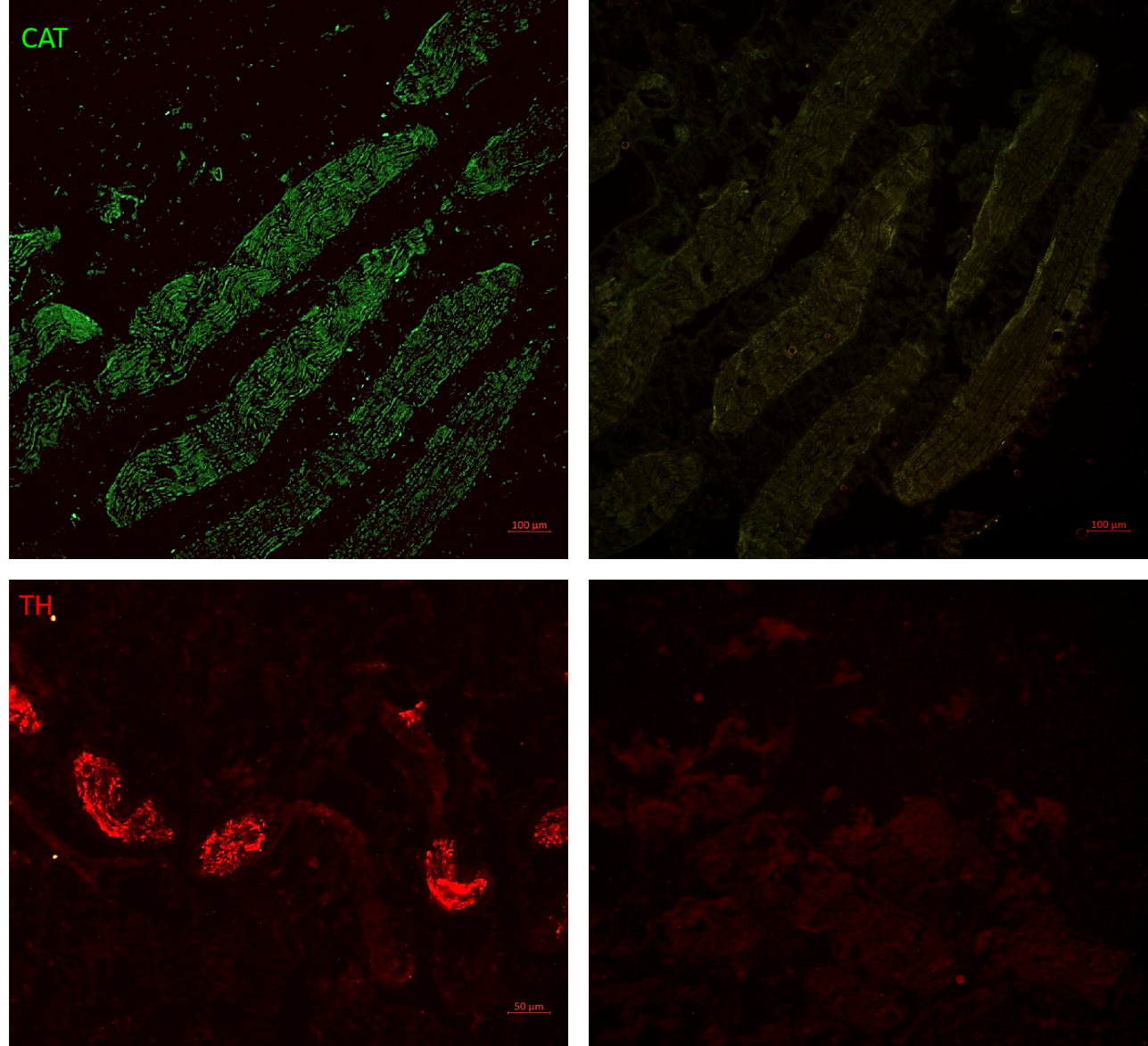


**Supplementary Figure 1.** Positive controls for tyrosine hydroxylase and choline acetyltransferase detection in porcine nerve tissues from one porcine heart. ChAT immunostaining was performed on a porcine vagus nerves (top left - green) and TH on porcine sympathetic trunk (bottom left - red) using the same immunohistochemistry technique we have used for all porcine tissues. The photographs on the top right and bottom right are negative controls without any antibodies, absent of immunofluorescence.

(ChAT = choline acetyltransferase; TH = tyrosine hydroxylase)
