# Supplementary figures and images for: Immunohistochemical characteristics of local sites that trigger atrial arrhythmias in response to high-frequency stimulation
Source: Europace. 2022 Oct 19;25(2):726–38. doi: 10.1093/europace/euac176 (PMC9935019; doi:10.1093/europace/euac176)

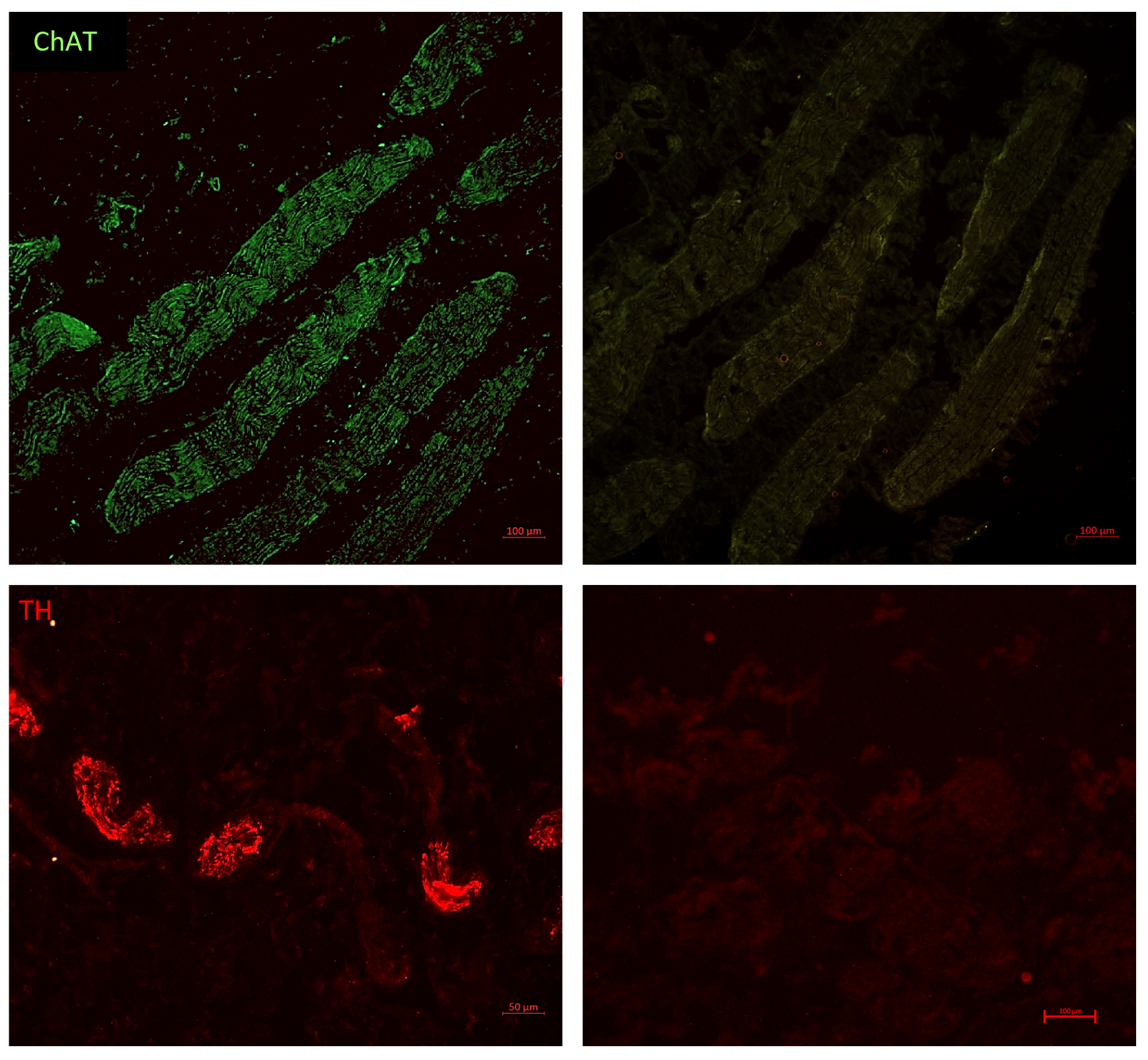

Supplement: euac176_Supplementary_Data [file euac176_supplementary_data.zip › EP Europace Supp Fig 1.tif]
